# Planning, Implementing, and Evaluating a Program to Address the Oral Health Needs of Aboriginal Children in Port Augusta, Australia

**DOI:** 10.1155/2012/496236

**Published:** 2012-04-22

**Authors:** E. J. Parker, G. Misan, M. Shearer, L. Richards, A. Russell, H. Mills, L. M. Jamieson

**Affiliations:** Australian Research Centre for Population Oral Health, University of Adelaide, 122 Frome Street, Adelaide, SA 5005, Australia

## Abstract

Aboriginal Australian children experience profound oral health disparities relative to their non-Aboriginal counterparts. In response to community concerns regarding Aboriginal child oral health in the regional town of Port Augusta, South Australia, a child dental health service was established within a Community Controlled Aboriginal Health Service. A partnership approach was employed with the key aims of (1) quantifying rates of dental service utilisation, (2) identifying factors influencing participation, and (3) planning and establishing a program for delivery of Aboriginal children's dental services that would increase participation and adapt to community needs. In planning the program, levels of participation were quantified and key issues identified through semistructured interviews. After 3.5 years, the participation rate for dental care among the target population increased from 53 to 70 percent. Key areas were identified to encourage further improvements and ensure sustainability in Aboriginal child oral health in this regional location.

## 1. Introduction

In Australia the term “Aboriginal” is used to denote the indigenous inhabitants of the mainland, Tasmania, and several other adjacent islands. “Indigenous” includes Torres Strait Islanders, who account for 6 per cent of the total Indigenous population, with 4% identifying as being of both Aboriginal and Torres Strait Islander origin [1]. The most recent information shows Aboriginal and Torres Strait Islander peoples represent 2.5 per cent of Australia's population, or 517,000 people [1]. In 2006, 32% of Indigenous people were living in major cities, 43% in regional areas, 9% in remote areas, and 15% in very remote areas [1].

### 1.1. Context

Historically Australian Aboriginal groups experienced lesser rates of dental caries than the non-Aboriginal population. In more recent times, this prevalence has reversed, particularly in children. Since the late 1970s there has been a marked overall reduction in dental caries rates in Australian children, for both the deciduous and permanent dentitions [2]. However, these improvements are not distributed evenly across the population and are not as evident in socially disadvantaged groups [3]. In contrast to the Australian average, during this same period, the oral health status of Australian Aboriginal children has deteriorated. Aboriginal children suffer rates of decay between 2-3 times higher than their non-Aboriginal counterparts, experience severe early childhood caries almost twice as often as non-Aboriginal children, and have a greater proportion of untreated caries in both the deciduous and permanent dentitions [4–6].

### 1.2. Setting

The town of Port Augusta is located 322 kilometres north of Adelaide, South Australia's capital city. In 2006 its population was recorded as 13,874 [7], of which the Aboriginal community represents 16.6% (2,303 persons), significantly greater than the Australian average. Port Augusta's Aboriginal community is culturally diverse, encompassing over 20 different language groups [8]. The Port Augusta Aboriginal community is serviced by the Pika Wiya Aboriginal Health Service (PWHS) whose mission is to “provide a culturally appropriate service to Aboriginal and Torres Strait Islander people, addressing preventative, promotive and curative aspects of health, which encourages our community to achieve greater dignity and quality of life equal with all Australians” [9]. Among a number of services offered at PWHS are child and maternal health, chronic disease management, immunisation, general practice, allied health services, and, since 2001, an adult dental service.

This project leading to the establishment of an Aboriginal children's dental service in the PWHS arose from issues identified from within the local community and by PWHS. Staff at PWHS raised concerns about an apparent discrepancy between the number of children enrolled in their child health program and the number of Aboriginal children enrolled in the School Dental Service (SDS) operated by the South Australian (SA) Dental Service and approaches to enrolling and recalling patients that may disadvantage Aboriginal children. In addition, staff at local public dental clinics reported lower participation rates for Aboriginal children in the SDS in comparison with non-Aboriginal children. Common exemplars included increased likelihood of missed appointments and Aboriginal children being lost to recall. The experience with patients attending the adult service suggested that poor rates of mainstream service utilisation were in part due to a dentist-centric and culturally unfriendly service, combined with transport and communication barriers [10]. We therefore suspected that similar factors were contributing to low service utilisation rates for Port Augusta's Aboriginal children. There were also anecdotal reports of Aboriginal children presenting with high levels of dental caries and requests from patients of the adult dental service to have their children also seen at PWHS. The apparent success and community acceptance of the adult model [10] led to growing demand from the Aboriginal community that a complementary children's dental service be offered in addition to the adult service within PWHS.

This paper describes the steps and early outcomes involved in the establishment of an Aboriginal Children's Dental Program (ACDP) in Port Augusta, SA. In particular the paper describes the rates of Aboriginal child dental participation in mainstream services prior to implementation of the ACDP through PWHS, factors influencing participation in the mainstream service, the framework for delivery of the Aboriginal children's dental service, and the participation rates and services delivered during the first three and a half years of the ACDP.

## 2. Methods

The partnerships formed in developing the existing adult dental service were utilised for this project to identify the issues regarding service participation and utilisation, construct a service model to address the issues identified, and then monitor and modify the service model as required to improve access, delivery, and service outcomes. 

### 2.1. Governance and Stakeholders

A Steering Committee was established comprising representatives from each of the key stakeholder groups: PWHS, the SA Dental Service, the University of Adelaide School of Dentistry and the Spencer Gulf Rural Health School. Two members of the steering committee also consulted with the PWHS Board of Management who gave feedback in the planning stages and were responsible for final approval before implementation of the Program. Comprised of representatives from the Aboriginal community, the Board of Management is the peak body which governs the delivery of PWHS services and programs. The members of the Board of Management and Aboriginal Health Workers from PWHS are considered advocates for their community, and throughout the planning and implementation of this project provided a means of ensuring community feedback was incorporated.

### 2.2. Ethical Approval

Approval to undertake this work was obtained from the Pika Wiya Health Service Board of Management. Approval was also granted by the SA Dental Service for access to school service statistics for children in Port Augusta. 

### 2.3. Service Utilisation

The level of participation of Aboriginal children in the School Dental Service (SDS), offered by the SA Dental Service, was quantified by cross-referencing the names of Aboriginal children enrolled in schools in Port Augusta with those listed on the mainstream SDS database in Port Augusta. The SDS provides dental care for children up to the age of 18, employing dental therapists and dentists. Children are provided with appropriate enrolment information when they enrol in preschool or school, aged from three and a half years. Data were analysed to determine the proportion of children with active or nonactive consent status. Children with an active consent status had been enrolled by their parents in the service, had attended for an examination, and not failed to attend appointments. Nonactive children included those not enrolled and those previously enrolled who had failed to attend appointments and not responded to clinic contact. The percentage of Aboriginal children listed with active consent status in Port Augusta in 2001 was compared to data from the South Australian Dental Service for all children in the state and specifically to the Port Augusta region for 2000. Data for Aboriginal students was obtained for 2 out of 3 preschools, all primary schools and the two high schools in Port Augusta. Data for preschool and primary school children were combined to determine overall participation rates. Comparison data after implementation of the new ACDP was obtained from both the mainstream SDS database and the PWHS database.

### 2.4. Information Gathering through Informal Interviews

Advice was sought through informal interviews from key staff from Port Augusta's SDS and PWHS to gather information about issues needing to be addressed by the proposed ACDP as well as to identify potential focus areas for service delivery. In particular, information was collected regarding possible reasons for lack of participation and challenges for current SA Dental Service staff in providing services for Aboriginal children. The four staff working at the local SDS and three PWHS staff were interviewed informally in the work environment, individually as well as in a group setting. Questions used to focus the discussion included

1. *What are the challenges you have faced in providing services for Aboriginal children?*
2. *What are the reasons you believe Aboriginal children may not attend appointments made?*
3. *What issues do you believe contribute to poor utilisation of services by Aboriginal children?*

Notes were taken during the interviews by the facilitator and key points confirmed with interviewees prior to concluding the interview. In order to refine issues and challenges for the current service and for the proposed service, the findings from the first round of interviews were grouped and summarised under key themes and discussed again with original interviewees. This process was repeated until there was consensus on the issues that would need to be addressed in a proposed new dental service delivery model.

## 3. Findings

### 3.1. Consent Status

In Port Augusta in 2000, there were 2839 preschool and primary school students eligible for the SDS. There were a total of 706 Aboriginal children enrolled in schools in Port Augusta, of which 537 (76%) were preschool and primary school students, and 169 (24%) were high school students. Of the Aboriginal children in Port Augusta, 316 (58.8%) of preschool and primary school children had an active consent status compared with 60 (35.5%) of high school students (Table 1). The overall consent rate for Aboriginal children in Port Augusta, combining preschool, primary school, and high school children in Port Augusta was 53.0%. The active consent rate for non-Aboriginal preschool and primary school children in Port Augusta was 85.7% (Table 1). These figures compare with a reported active consent status of 76.1% for preschool and primary school students and 49.4% for high school students for SA overall in 2000 [11]. The average combined (Aboriginal and non-Aboriginal) participation rate for preschool and primary school students in 2000 for Port Augusta was 77.0%.

### 3.2. Factors Influencing Participation

Informal interviews with SDS and PWHS staff identified a number of key issues influencing participation rates. These included a high rate of failure to attend appointments, attendance patterns for relief of pain rather than follow-up care, difficulty contacting parents and guardians, and difficulty communicating effectively with parents and guardians (Table 2). Pika Wiya Health Service staff identified additional issues including difficulties with transport, not receiving appointment cards, lack of knowledge of the importance of dental care, and a lack of Aboriginal staff in the dental service. 

### 3.3. Framework for the Delivery of Aboriginal Children's Dental Program

The commitment and shared responsibility of key stakeholders were considered crucial to successful implementation of the Program. The South Australian Dental Service funded the clinical service components, including staffing of a dental therapist and dental assistant, consumables, and maintenance of clinical equipment. The PWHS provided funding for an Aboriginal Primary Health Care Worker and transport officer, provided office space, nonclinical equipment, and all on-costs. The Spencer Gulf Rural Health School provided scholarship funds for a postgraduate dentist already working with the adult service to manage the Program, provide clinical support and plan and deliver health promotion components. The University of Adelaide School of Dentistry continued to provide an advisory role, support and mentoring for the postgraduate dentist, and provided an avenue for development of future research projects.

A service model that addressed the key challenges and barriers identified was established to meet the goal and objectives of the project. The service model was reviewed regularly with dental service staff, staff involved in other programs within PWHS, Aboriginal Education Workers, staff at the schools, and PWHS and SA Dental Service management. Modifications to the model were implemented as potential improvements were identified. In particular, the service was to be given the freedom of adapting to feedback from the community it was designed to serve.

Whilst the model planned to focus on primary health care, implementing early intervention and health promotion strategies, it was acknowledged by stakeholders that this would take time to achieve, and the prime focus was therefore to be a service model that encouraged and facilitated participation by the local Aboriginal community. 

The overall goal of the ACDP was to improve the oral health of Aboriginal children in Port Augusta. Specific objectives included (1) to provide a culturally friendly children's dental service, (2) to ensure that all school-age Aboriginal children have a comprehensive examination, (3) to ensure each child completes the course of treatment arising from that examination, (4) to establish and manage a system of recalls, (5) to provide an emergency service, (6) to ensure a strong focus on community health promotion, and (7) to increase the number of Aboriginal children accessing routine and preventative dental services.

Stakeholders agreed to implement a School Dental Program from PWHS (the ACDP) as a pilot project in the first instance. Commencing in February 2002, the ACDP took the following form for the initial 12 months

1. Operated from within the existing PWHS Dental Clinic
2. Operated one day each week
3. Utilised a South Australian Dental Service-employed dental therapist and dental assistant
4. An Aboriginal Health Worker organised patient appointments and liaison
5. The service was supported by a dentist who managed Program implementation and provided clinical support
6. Key service statistics were collected through routine case notes and data entry
7. School-based health promotion Programs were instituted and conducted by the Aboriginal Health Worker and dentist together with other clinic staff as appropriate.

Key differences between the ACDP and the existing mainstream SDS are described in Table 3.

### 3.4. Health Promotion

Health Promotion activities in the first 12 months of the Program included the Aboriginal Health Worker and dentist providing an education session for all classes in five of the six Port Augusta primary schools and the kindergarten with the greatest number of Aboriginal enrolments. School-based health education sessions focussed on providing health information for children and staff, as well as promoting the service and ensuring children were familiar with staff and the concept of dental visits and treatment. Staff also participated in broader health promotion activities and community events.

From 2003, the Aboriginal Health Worker and dentist worked with the dietitian at PWHS to deliver joint health promotion activities, such as school-based activities, demonstrations of healthy cooking and snack alternatives, and involvement in community health events. In 2004, four dental students from the University of Adelaide School of Dentistry helped to deliver a workshop for Aboriginal Health Workers on oral health. In addition, they developed a series of simple information sheets for Aboriginal Health Workers and patients on the importance of oral health for pregnant women, people with diabetes, and people with heart disease. In 2005, six dental students were also involved with developing health promotion resources aimed at different age groups. Further health promotion programs have been developed through research partnerships with the University of Adelaide School of Dentistry but are beyond the scope of this paper. 

### 3.5. Program Adaptation

Consistent with the aim of adapting to community needs, the Program continued to evolve. Enrolment of children into the SDS or the Program normally occurs by the parent once information about dental services is provided by the School. In order to assist in enrolment of Aboriginal children in the SDS or the Program, contact was maintained with Schools. Strategies were explored to ensure that privacy was maintained whilst acknowledging the need to address and support the particular health needs of some students and families. For example, a school representative visited the clinic and worked with the Aboriginal Health Worker to identify students who needed to be contacted, without either the school or clinic releasing students' details.

Due to funding problems, there were periods where the ACDP needed to function without an Aboriginal Health Worker. The loss of this employee, as well as transport staff accepting more permanent positions elsewhere, contributed to high staff turnover for the program. As the Aboriginal Health Worker was the key link with the community, other ACDP staff needed to take on additional roles and attempt to fill aspects of the position in their absence. This proved difficult, stretching staff already functioning in demanding roles. Staff also believed that the lack of continuity of an Aboriginal Health Worker with the program restricted the development and growth of the ACDP, potentially affecting enrolment and participation rates as well as service productivity.

After a full examination and determination of an appropriate treatment plan for the patient, an appropriate interval for a recall appointment is determined based on an assessment of probable disease risk. In late 2004 and 2005, it was noted that the planned appointing of recalls was behind schedule, thought to be as a result of the high treatment needs of individual patients and the challenges with maintaining anticipated productivity whilst managing failed appointments. To ensure that the recall times did not continue to extend, the dental therapist began making appointments for clients on the current recall list at the scheduled time, whilst also trying to clear the backlog of recall appointments. The dental therapists reported that patients recalled at the scheduled time seemed to have fewer treatment needs than when first entering the service and fewer needs than those who had waited longer for the recall visit.

### 3.6. Participation Rate

The establishment of the ACDP saw an increase in the overall participation rates of Aboriginal children in dental services in Port Augusta as well as a shift in the distribution of enrolment from the mainstream SDS to the ACDP offered at PWHS (Table 4).

The number of Aboriginal children enrolled in the ACDP at the PWHS in October 2005 (after approximately 3 and a half years of operation) was 679, and the number of children on the recall list was 438. This indicates that 64.5% of the children attending for comprehensive examination and treatment planning were then placed on the appropriate recall list (Table 4). The number of Aboriginal children enrolled in the Australian Dental Service mainstream SDS was 137, with 31 children enrolled in both the mainstream SDS and the ACDP, giving a total of 785 Aboriginal children enrolled in either the mainstream SDS or ACDP in October 2005. The proportion of children enrolled and on recall was 544 (69.3%). This figure has been used as a conservative estimate of the participation rate in dental programs, the closest in definition to the “active consent rate” criterion used with the mainstream SDS, giving an increase in participation rates from 53% in 2002 to 70% in 2005.

Regular cross-checking of names of children enrolled in the mainstream SDS ensured that duplication between the dental programs was minimised. Once it was confirmed which clinic a family wished to attend, the child's name was removed from the alternative clinic's contact list.

### 3.7. Service Statistics

**Figure 1:**
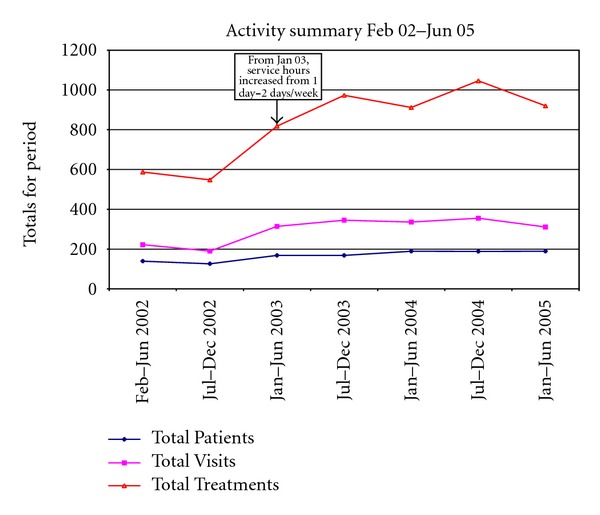


From February 2001 to June 2005, a total of 591 clients were seen over 2074 visits (Figure 1). Participation rates steadily increased over the first year of operation, stabilising after 24 months. Given early demand for appointments and procedural services, it became necessary for the clinic to open an additional day each week, opening 2 days per week from late January 2003.

**Figure 2:**
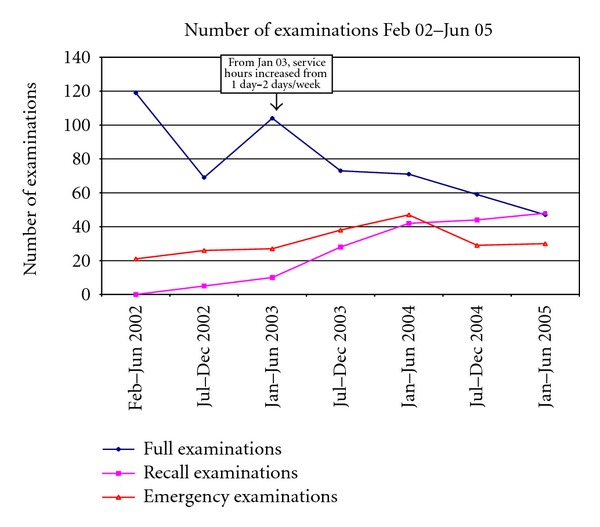


From February to June 2002, 119 new patients had a full examination, including a treatment plan (Figure 2). Following the increase in the number of operating days in January 2003, the number of new patients having a full dental examination decreased steadily, indicating that over time the backlog of clients was decreasing. However, as a direct consequence, the number of patients attending for recall examinations steadily increased, reflecting the increase in patients who completed their initial course of treatment and were attending for a “check-up” or recall examination.

**Figure 3:**
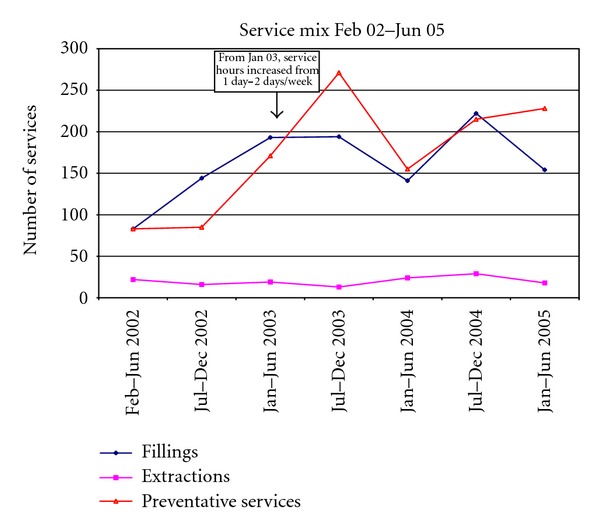


The number of emergency examinations remained reasonably constant, averaging 31 emergency examinations in each half yearly period (Figure 2). Emergency examinations indicate that a patient attended for a specific concern only and may include patients new to the service, patients who attend outside of their planned recall with an urgent problem, or patients not normally enrolled in the service. The number of extractions remained reasonably constant throughout the operation of the service, averaging 20 extractions in each half yearly period (Figure 3). The number of restorative procedures performed increased initially and then plateaued, again demonstrating clearing of a backlog of required procedures. The number of restorations placed in each period levelled off at a mean of 162 from January 2003 onwards.

**Figure 4:**
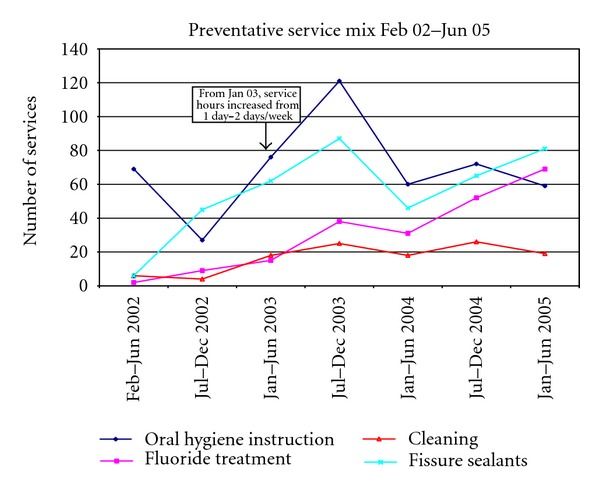


The number of preventative services provided in each six-month period increased from 85 in Jul-Dec 2002 to 271 in Jul-Dec 2003, and 228 in Jan-Jun 2005 (Figure 4). The mix of preventative services demonstrated a hump pattern, changing throughout the service's operation. Fluoride treatments increased steadily whilst the number of cleaning services remained constant from Feb 2002. While oral hygiene instruction services saw a dramatic increase from Jun-Jul 2002 to Jul-Dec 2003, the number of oral hygiene instruction services remained stable outside that period. The number of fissure sealants, placed to prevent dental caries in the pits and fissures of teeth, mimicked the pattern of oral hygiene instruction from Jul 2002 to Dec 2004. 

**Figure 5:**
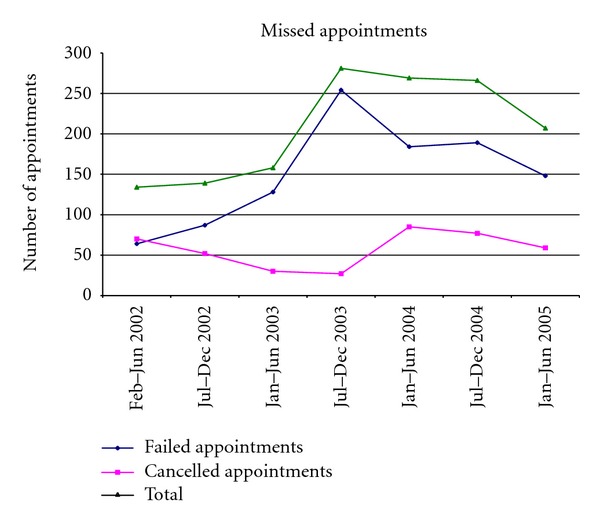


The number of cancelled and failed appointments increased in the first four periods, peaking in the period Jul-Dec 2003, with a total of 281 missed appointments (Figure 5). Failure to attend appointments accounted for the majority of missed appointments across all but the first period.

## 4. Discussion

This paper seeks to describe the establishment and impact of an Aboriginal Children's Dental Program in Port Augusta, South Australia, a large regional town with an above State average Aboriginal population. The imperative for the service arose from concerns of underutilisation of the mainstream School Dental Service by Aboriginal children by mainstream service staff as well as similar concerns by the local Aboriginal health service and concerned community members. 

### 4.1. Success Factors

The success of the service is demonstrated by the increased enrolments and increased participation rates compared with figures under the mainstream only service. Just over half the Port Augusta-based Aboriginal child population was enrolled in the mainstream SDS prior to implementation of the tailored ACDP, compared with 70 percent being enrolled, having had an examination and recall assigned, after three and a half years of the program's operation.

There were a number of likely characteristics of the new service that may have been responsible for its apparent success. Arguably, the opportunity for the local community to participate in the development of the children's dental services was a key factor. A quote from the rural health policy framework Healthy Horizons Outlook confirms this premise:

*“participation by individuals, communities and special groups in determining their health priorities should be pursued as a basis for successful programs and services to maintain and improve their health.” [12]*

The document further comments that

*“social community participation in rural health service development has been argued to result in more accessible, relevant, and acceptable services.” [12]*

Key findings from the interviews concerning factors affecting low participation rates for the mainstream SDS were that the service was not culturally friendly—including not employing Aboriginal staff—not integrated with other services, and that the community did not have a full understanding of the importance of oral health or of the benefits of preventative services. The importance of training and employing Aboriginal staff and in developing trust relationships between health professionals and Aboriginal communities was also emphasised by Pacza et al. in their description of the development of a culturally appropriate basic preventative oral health delivery program at a community level in rural and remote Western Australia [13].

In our project, the fact that there was an opportunity for the community to provide input into the design and operation of the service in itself was an important initiative of those planning the service. Seeking community input and incorporating community feedback and advice into the service design provided a sense of community ownership that saw the service better accepted and utilised than the previous model. Similar findings have been described by other authors, highlighting the importance of establishing both formal and informal relationships between community and health services and locating services in a culturally friendly environment [14].

Our information seeking approach encouraged communal problem identification, sharing of probable causes and proposal of solutions, by a range of stakeholders. Our informal interviews were useful in determining multiple factors influencing participation. Having informal and group discussions helped interviewees to feel comfortable and therefore enabled open discussion. It was interesting to note the similar perspectives of those representing different stakeholder groups, and the consistency with factors identified by other sources [15].

Further underpinning the success of this program is the fact that it is incorporated into an existing Aboriginal Community Controlled Health Service, enabling a more culturally appropriate approach to service delivery and community ownership of the program. This is consistent with the recommendations made from the National Aboriginal and Torres Strait Islander Oral Health Workshop in 2002 [15]. Furthermore, having an Aboriginal Health Worker at the centre of the program to liaise with and support patients, introduce them to the ACDP, and provide cultural advice for non-Aboriginal staff has enabled many of the key factors identified through informal interviews to be addressed. The involvement of Aboriginal Health Workers or other similar community liaisons has been recognised as a key success factor in other primary care service programs and in research [16–18].

The enthusiasm and commitment of staff was crucial in the implementation and success of the Program. The cooperation of different organisations, as represented by the working party, ensured that the expertise of each organisation was utilised to provide desirable outcomes. An example of this is the funding model, whereby the South Australian Dental Service paid for clinical service provision, PWHS paid for support staff and on-costs, and the Spencer Gulf Rural Health School paid for the dentist to oversee implementation of the project, to manage the service and to provide clinical support.

The foregoing elements illustrate two of the four conceptual approaches of community participation described by Taylor et al., namely, the *community empowerment approach* and the *developmental approach*. The former seeks to empower and support communities, individuals, and groups to take greater control over issues that affect their health and wellbeing and incorporates notions of personal development, consciousness-raising, and social action. The latter is underpinned by principles of social justice and describes health and social care development as an interactive, evolutionary process, embedded in a community of place or interest and where local people, in partnership with professionals, and have a role in decision-making and in achieving the outcomes they consider important [19].

The fact that in October 2005 there were still 137 Aboriginal children enrolled in and attending the mainstream SDS that indicates that, for a portion of the population, the School Dental Service is preferred. Further drawing on the partnership between the ACDP and the SDS, if patients enrolled with the SDS failed to attend appointments and failed to respond to recall notices, their names and records were given to the ACDP for follow-up, to ensure that children were given all chances of participation. In some instances the family situation may have changed—they may have moved house or moved out of town altogether—making contact by mainstream clinic staff very difficult. PWHS staff have the contacts and community knowledge to determine reasons involved in nonattendance that may be beyond the capacity of the mainstream service. This reinforces the need for programs to be flexible and adapt to the potentially changing needs of the community and individual patients, and to utilise local staff with an understanding of and connection with the local community [20].

### 4.2. Challenges

The ACDS at PWHS attempted to address the issues identified through the interviews, some of which were more easily addressed than others. For example, the provision of transport for children to and from school to the dental service was effective, but parents and carers were less likely to accompany children when transport was offered. This limits opportunities by clinic staff to provide oral health information to parents or guardians that encourages preventative oral health practices at home and among other family members and that reinforces the importance of attending follow-up appointments.

Despite significant efforts there were still a large number of children who were registered with the clinic but had not attended for a full examination and subsequent treatment. For the purposes of evaluating changes in participation before and after the ACDS was implemented, the number of children who were enrolled and had an assigned recall was used. This may have resulted in a somewhat conservative estimate of the improvements in participation. The difference between the number of children enrolled with the PWHS Program and the number of children on recall indicates that 241 children were registered but had not had a full examination, treatment plan, and recall assigned. Possible reasons include (1) patient attended for a specific concern only; (2) patient returned enrolment form but was never appointed due to an error in appointing procedures; (3) patient appointed but never attended; (4) unable to contact parents/guardians for consent for transport; (5) patient moved and not contactable; (6) failed to assign a recall when examination conducted; (7) unable to contact parents for consent to complete the treatment deemed required at the examination appointment. Strategies were developed to address these possibilities, including better processes for enrolling and appointing patients, database searches to ensure that all patients were on recall, and more home visiting and contact with families. The impact of these additional strategies is still being assessed.

The high rate of failure to attend scheduled appointments and cancellations demonstrates the difficulty in predicting attendance and planning comprehensive care delivery in this patient group. The unpredictable nature of patient attendance had also been an issue in the adult service [10], and whilst often due to cultural or family obligations [21], it has a significant impact on the clinical productivity. There were several strategies explored and implemented for managing the high number of failure to attend appointments. These included double booking multiple patients, appointing children in school groups and contacting the schools on the morning of the appointment to check which patients are at school on that day, and contacting patients and parents the day prior to their appointment. These approaches, however, did not successfully address the issue. Involvement of parents in the ACDP was crucial to ensure that informed consent for treatment was gained, that parents understood the importance of their child's ongoing dental care, both in terms of dental visits and treatment but most importantly home care. Encouraging parents to attend appointments with their children was an ongoing issue, requiring continued attention and application of various strategies to improve parental involvement.

### 4.3. Limitations

It is important to recognise the limitations of this project. Firstly, this was a project designed specifically to address a local community issue, with a small target population, limiting the transferability to other populations. Secondly, this was designed as a community participatory service project to respond to local needs, working with local community members, with mainstream public dental service providers, and with the local Aboriginal health service, in a real-world environment rather than in a controlled research environment. As a consequence, a number of day-to-day factors affecting service uptake (e.g., sickness, funerals, and cultural events) were beyond our control. Availability of data was necessarily constrained by resources and was limited to data collected as part of routine public dental health practice in SA. Accordingly, comparison of service utilisation rates with data outside the time period of the service of more broad SA data is not presented, nor are disease rate comparisons although some has been reported elsewhere [22].

In contrast, it is noteworthy that many Indigenous communities in Australia and overseas are small and face similar issues in terms of general and oral health outcomes [23, 24] and have the need to work with local agencies to develop suitable programs and services for their communities. The community participatory framework and learnings derived from this project may serve as a basis for other communities.

### 4.4. Program Sustainability

From July 2005 to June 2011 the core components of the ACDP have been maintained, proving that a sustainable service delivery model was developed. During this time, there have been continued modifications to the program in order to ensure that the community needs are met as well as to maintain sustainability and an appropriate spread of resources across the region. Crucial to the continued delivery of the service has been the role of the SA Dental Service continuing to fund and manage the clinical service, and PWHS continuing to fund and support an Aboriginal Health Worker position. The partnership with the University of Adelaide School of Dentistry continues, enabling students to participate in elective projects that have enhanced the health education and health promotion components of the program. Since 2005 there has been a decrease in time and resources dedicated to the health education and health promotion aspects of the program. However, continued partnerships with the University of Adelaide School of Dentistry and the Spencer Gulf Rural Health School have enabled research projects focussed on health promotion interventions to be implemented for the community working in partnership with the clinical service providers.

## 5. Conclusion

A culturally appropriate dental program for Aboriginal children in Port Augusta was successfully established through a partnership with the South Australian Dental Service, PWHS, the Spencer Gulf Rural Health School and University of Adelaide School of Dentistry, building on a recently established service for Aboriginal adults. The new dental program for children which incorporated Aboriginal Health Workers and health promotion activities resulted in an increase in participation rates from 54% to 70% of eligible children. While this is a pleasing outcome, a number of challenges still remain to achieve the goal of improving dental outcomes for Aboriginal children in Port Augusta.

## Figures and Tables

**Table 1:** Consent rates for Aboriginal children in Port Augusta with comparisons to state-wide data.

School type (number of schools included)	Number of Aboriginal students (range for individual schools)	Pt Augusta Aboriginal active consent rate (range for individual schools)	SA 2000	Port Augusta non-Aboriginal calculated active consent
Pre and primary school (8)	537 (4–129)	58.8 (0–75.9)	76.1%	85.7%
High school (2)	169 (16–53)	35.5 (30.7–81.3)	49.4%	Not available

**Table 2:** Key issues and challenges identified through informal interviews.

Key issue/challenges	Associated issues/barriers identified
Difficulty contacting patients	Frequent change of address
	Irregular use of letterboxes
	Appointment cards returned to sender
	Patients moving towns at a high rate, sometimes leaving Pt Augusta and returning months later—clinic not notified
	Patients not on telephone or frequent change of number
	Children changing schools
	Nonattendance at school
	Schools unable to release contact details
	Patients may use more than one surname

High rate of failure to attend appointments	Difficulties with transport
	Priority placed on oral health in combination with other physical, social, and emotional well-being issues of individuals and families
	Wednesdays and Thursdays are “money days” when pensions are paid, bringing with it other priorities for adults
	Lack of understanding of the importance of dental care

Attendance patterns	Attend for relief of pain
	Nonattendance for routine and preventative care
	Lack of understanding of the importance of preventative care in contrast to relief of pain

Consent issues	Patient may be residing with someone who is not parent or legal guardian
	Difficulty contacting parent or guardian when treatment decision needs to be made
	Cultural differences and rules in regard to consent not understood and accepted by mainstream policies

Difficulty communicating with parents and guardians	Parents and guardians often seem uncomfortable in the dental clinic
	Parents seem uncomfortable communicating with dental service staff
	Lack of understanding of dental disease, prevention and importance of dental care
	Different approach to and understandings of treatment decision making (different between staff and patients/parents)
	Lack of Aboriginal staff in the mainstream dental clinic

**Table 3:** Key differences between ACDP and the mainstream SDS.

Issue	Aboriginal child dental program	Mainstream school dental service
Location	The clinic operated from within PWHS, a service that the local Aboriginal community was accustomed to accessing for whole of health and health-related services	Mainstream clinic, used by non-Aboriginal and Aboriginal clients

Integrated	Into the health service as part of the holistic philosophy of Aboriginal Health Care	Not part of any other health service

Health promotion	Staff were involved in health promotion initiatives in schools and through community events	No specific health promotion programs targeting Aboriginal children or families

Staff	An Aboriginal Primary Health Care worker was the key contact and liaison for the community and clinical staff, providing community links, contacts, information, and cultural support. Over seventy percent of the staff at PWHS are Aboriginal and are representative of the diverse language groups accessing the service	No Aboriginal staff

Transport	A transport officer was employed to transport children and parents to and from dental appointments, to the chemist and specialist services	No specific transport services available for Aboriginal clients

**Table 4:** October 2005 enrolments.

	Mainstream School Dental Service	PWHS	Dual enrolment	Total School Dental Service enrolment	As a percentage of school enrolments
Enrolled	137	679	31	785	101%
On recall	137	438	31	544	70%
